# A comparison of physiological intensity and psychological perceptions during three different group exercise formats

**DOI:** 10.3389/fspor.2023.1138605

**Published:** 2023-03-31

**Authors:** Jinger S. Gottschall, Bryce Hastings

**Affiliations:** ^1^ Department of Integrative Physiology, University of Colorado, Boulder, United States; ^2^ Health & Environmental Sciences, Auckland University of Technology, Auckland, New Zealand

**Keywords:** group fitness classes, exercise enjoyment, exercise satisfaction, exercise intensity, virtual fitness

## Abstract

**Introduction:**

Past research highlighted that group fitness is an ideal format to meet exercise prescription guidelines. To add, a group enhances exertion, enjoyment, and satisfaction. In the last five years, streaming (live classes on screen with other participants visible) and on demand (pre-recorded classes on screen without other participants visible) formats have grown in popularity. Our goal is to compare the physiological intensity and psychological perceptions of live group, live streaming, and non-live on demand classes. We hypothesize that live classes will have the greatest cardiovascular intensity, enjoyment, and satisfaction followed by streaming and finally on demand.

**Methods:**

Fifty-four adults between 18–63 years, who regularly participate in group fitness classes, recorded their heart rate with a chest transmitter during a mixed-martial arts cardiovascular class on consecutive weeks in random order. We calculated the mean, identified the max, and extracted the top 300 values (5 min) for comparison between conditions.

**Results:**

Following each class, the participants completed an online survey to evaluate their rate of perceived exertion, enjoyment, and satisfaction. Confirming our hypothesis, mean class heart rate and mean heart rate for the five minutes at the highest intensity were 9% greater during the live group format compared to both live streaming and non-live on demand (all values, *p* < 0.01). However, there was no difference in any heart rate variables between the streaming and on demand formats. Also, rate of perceived exertion, enjoyment, and satisfaction were all significantly greater during the live session compared to the home collections (all values, *p* < 0.05).

**Discussion:**

Streaming and on demand group fitness formats are viable options for meeting exercise prescription guidelines. But physiological intensity and psychological perceptions were greater during the live class format.

## Introduction

Physical activity, even a singular bout, is correlated with an assortment of positive physiological and psychological health benefits such as improved sleep, cognition, and insulin sensitivity, in addition to, reduced anxiety and blood pressure (1). Despite these beneficial outcomes, many individuals remain inactive. For example, in the United States, recent data from the National Health Interview Survey indicate that less than 60% of adults are meeting the higher 300 min per week of cardiovascular exercise guidelines (2).

Group fitness is one method utilized to promote physical activity across a wide array of people, in both healthy and clinical populations. Previous reviews have reported impressive results with respect to the physiological and psychological benefits of exercising in a group (3–5). In fact, prior to the coronavirus pandemic in 2020, the American College of Sports Medicine ranked group fitness as one of the top three fitness trends (6). One year later, online live streaming was the number one trend. Currently, online live streaming paired with non-live on demand classes was still part of the top ten fitness trends but dropped to number nine while group fitness did not make the list.

Our research highlights many of the reasons why group classes have been so popular in the past (5, 7–9). We employed both surveys to assess groupness, effort, and positive affect as well as heart rate monitors to evaluate cardiovascular exercise intensity. For groupness, we asked if the participants perceived the members in the class as a single entity and if there were roles as well as typical behaviors of the members. During classes in which perceptions of groupness were greater, the participants reported more exertion and greater positive affect. With respect to intensity, group environments yielded a higher heart rate during cardiorespiratory classes as well as heavier weight selection during strength classes. Also, exercising together, with the ability of the instructor to interact with verbal encouragement and facial expressions, led to feelings of enjoyment and satisfaction.

However, joining a live group exercise class, especially during a pandemic, is not always a feasible option. In the last five years, streaming (live classes on screen with other participants visible) and on demand (pre-recorded classes on screen without other participants visible) formats have grown in popularity. Over 85% of gym members complete regular exercise sessions at home and these numbers are only increasing with the repercussions of the global pandemic (10). To add, survey data of over 2,000 adults and adolescents, established that individuals who use digital platforms for physical activity were more likely to meet both the moderate-to vigorous cardiovascular recommendations as well as the muscle strengthening guidelines as published by the World Health Organization (11, 12). In terms of potential explanations, Silva-Jose et al. (13) conducted interviews with women who regularly completed exercises at home and their reasoning for regular participation was increased availability to complete a class, in short, flexibility of schedule and convenience.

A recent study by Eckmann et al. (14) compared various cardiovascular, body composition, and muscle endurance variables between groups who completed live and on demand formats. They reported that both formats improved health related outcomes after a 10-week protocol of a fusion class three days a week. With this recent surge in group fitness options, it is also critical to assess the differences in the offerings to provide recommendations for the ideal format and promote the options with the greatest opportunity for adherence. Therefore, our goal is to compare the physiological intensity and psychological perceptions of live group, live streaming, and non-live on demand classes. We hypothesize that live classes will have the greatest cardiovascular intensity, enjoyment, and satisfaction followed by streaming and finally on demand.

## Methods

Fifty-four adults (44 ± 8 years, 8 men), from five different geographic locations in the United States and who completed a minimum of five hours of planned exercise per week volunteered to participate. Participants were mostly Caucasian (75% Caucasian, 19% African American, 6% identified as other). Sixty-seven percent of the participants had been completing group fitness with the experimental format for over three years while only 16% had less than a year of experience.

The total study duration was four weeks, one familiarization week to test the heart rate monitors and introduce survey questions, and three experimental weeks. The conditions were live group, completed at a gym or studio, with the instructor and other participants physically present; live stream, completed at home, with the instructor and other participants visible on a screen; and non-live on demand, with the instructor previously recorded. The participants collected heart rate data during each class with an H9 chest transmitter (Polar Electro Oy, Kempele, Finland) with Bluetooth connection to their mobile device using the Polar Beat app. After each exercise session, they exported their data from the Polar Flow website and completed an online survey on their computer.

All classes were the same Les Mills BODYCOMBAT release, with the experimental conditions (live, streaming, on demand) completed in random order for each location. The release is a pre-choreographed 51-min routine completed with a musical playlist. Thus, each class had the identical sequence of exercises completed with identical timing. The live group and live stream in a particular geographic location were instructed by the same master trainer. The on demand condition was a video from which the master trainers learned the choreography.

The Polar H9 recorded heart rate data each second, yielding 3,060 data points per class. For the three conditions, we matched the start time and clipped the files to ensure each session included the same number of heart rate entries. We subsequently calculated the mean, identified the max, and extracted the top 300 values (5 min) for comparison between conditions. Because the class was choreographed to music with the same timing and repetitions for each exercise, we could match the intervals between conditions to ensure they were equal.

We collected the subjective class data electronically (Qualtrics, Washington & Utah, United States) with a survey link within 1 h of completing the class. The questions included rate of perceived exertion, class and instructor satisfaction, effort, enjoyment, positive challenge, concern about performance and execution, fatigue, amount of activity, and instructor ability as well as encouragement. Each of the survey items was gathered using a slider with a scale of 1–7 except for rate of perceived exertion which was a scale from 6 to 20. We calculated the mean and standard deviation of each individual item from the three experimental class formats for statistical comparison.

Rate of perceived exertion (RPE) was measured using the Borg scale (15) and a fatigue item. We provided the following instructions: “Rate how hard you had to exert yourself during the exercise class you just completed. Focus on your total feeling of exertion. Do not focus on just one factor such as shortness of breath or leg pain.” The participants rated their perceived exertion on a 6 (no exertion at all) to 20 (maximum exertion) scale. Fatigue was assessed using “I feel very fatigued right now.” Participants responded using a 1 (not true of me at all) to 7 (extremely true of me) scale.

Satisfaction with the class was assessed using five items. The initial two items included “I was satisfied with the class,” and “I was satisfied with the instructor.” The final three items included effort, enjoyment, and challenge; “I put a lot of effort into this class,” “I enjoyed this class,” and “I viewed this class as a positive challenge.” Participants responded using a 1 (not true of me at all) to 7 (extremely true of me) scale.

Perceived competence was measured using two items similar to Maher et al. (9). One item represented task-referenced competence, “I believe I completed the exercises today the way they should be done,” while the other represented self-referenced competence, “I believe I improved today compared to my past performances in the class.” Participants rated each of these items on a 1 (not true of me at all) to 7 (extremely true of me) scale.

Instructor behavior was assessed using three single-item measures (16). Performance and encouragement were assessed with the items, “The instructor helped me focus on performing as well as I can perform,” “The instructor helped me to focus on not performing below my ability,” and “The instructor encouraged me.” Participants rated these items using a 1 (strongly disagree) to 7 (strongly agree) scale.

We analyzed heart rate and survey variables using a repeated measures ANOVA to distinguish differences between formats. We completed Scheffe's *post hoc* tests when necessary (*p* < 0.05). All data are presented as mean ± standard deviation.

## Results

**Figure 1:**
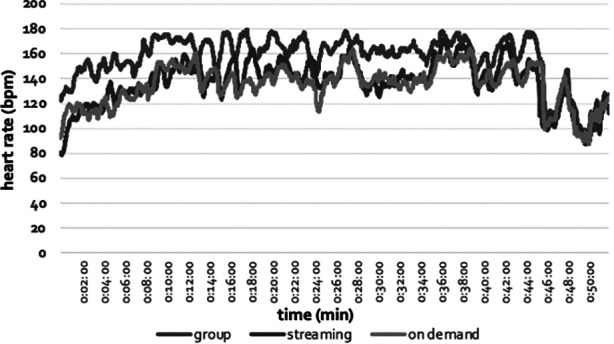
Example beat-by-beat heart rate data for a single participant.

**Figure 2:**
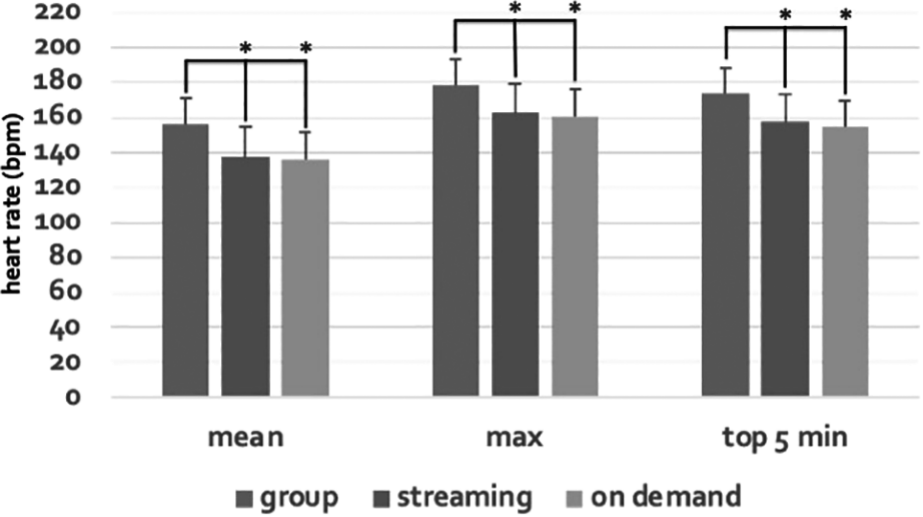
Mean class heart rate, max heart rate, and mean heart rate for the five minutes at the highest intensity during the live group format compared to both live streaming and on demand An * equals a statistical difference with live group (all values *p* < 0.001). In addition, there was no difference in any heart rate variables between the streaming and on demand formats.

The live group class heart rate variables were statistically greater than both the live streaming and non-live on demand classes. For example, mean class heart rate (group = 149 ± 14; streaming = 132 ± 14; on demand = 131 ± 14), max heart rate (group = 172 ± 13; streaming = 157 ± 15; on demand = 156 ± 16), and mean heart rate for the five minutes at the highest intensity (group = 167 ± 13; streaming = 151 ± 15; on demand = 150 ± 16) were 14%, 11%, and 10% greater, respectively, during the live group format compared to both live streaming and on demand (all values, *p* < 0.001, Figures 1, 2). In addition, there was no difference in any heart rate variables between the streaming and on demand formats. Similarly, rate of perceived exertion was 17% greater during the live group class (RPE = 18 ± 2) compared to the other two formats (both classes RPE = 15 ± 2; all values, *p* < 0.001).

**Figure 3:**
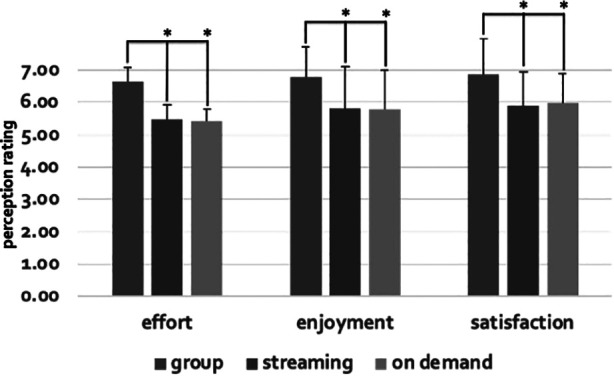
Mean class effort, enjoyment, and satisfaction during the live group format compared to both live streaming and on demand. An * equals a statistical difference with live group (all values *p* < 0.001). However, the participants were more self-conscious and concerned about their performance during the live group format.

With respect to the survey measures, in general, the positive affect variables were greatest during the live group class but there were also a few negative affect variables that were also highest in that condition (Table 1). Class satisfaction was 14% and 13% greater during live group compared to live stream and non-live on demand respectively (all values, *p* < 0.001, Figure 3). To add, effort, enjoyment, and positive challenge were a minimum of 12% greater during the live group class. However, the participants were 32% less self-conscious and 39% less concerned about their perception during the non-live on demand class compared to the live group (all values, *p* < 0.001).

**Table 1:** Survey items gathered using a slider with a scale of 1–7 except for rate of perceived exertion (RPE) which was a scale from 6 to 20. An * equals a statistical difference with LIVE group (all values *p* < 0.05).

	LIVE group	LIVE streaming	NON-LIVE on demand
RPE	18.18 ± 1.69	15.03 ± 1.89*	15.18 ± 2.07*
Class satisfaction	6.86 ± 0.38	5.89 ± 1.21*	5.98 ± 0.93*
Instructor satisfaction	6.95 ± 0.17	6.77 ± 0.43*	6.28 ± 0.85*
Effort	6.64 ± 0.45	5.48 ± 0.92*	5.42 ± 1.14*
Enjoyment	6.80 ± 0.45	5.84 ± 1.28*	5.79 ± 1.05*
Positive challenge	6.82 ± 10.39	6.03 ± 1.11*	5.94 ± 1.10*
Concerned performance	3.33 ± 2.25	3.60 ± 2.27	2.96 ± 2.10
Correct execution	6.42 ± 0.76	5.81 ± 1.77*	5.58 ± 1.24*
Comparison to past	5.71 ± 1.42	3.86 ± 1.68*	4.21 ± 1.61*
Fatigued	5.20 ± 1.80	4.31 ± 1.80*	4.44 ± 1.61*
Self-conscious	3.19 ± 2.13	2.58 ± 1.68*	2.16 ± 1.68*
Concerned perception	2.84 ± 1.69	2.26 ± 1.46*	1.73 ± 1.06*
Satisfaction activity	6.82 ± 1.69	5.68 ± 0.94*	6.06 ± 1.48*
Instructor helpful	6.74 ± 1.69	6.31 ± 1.16*	5.45 ± 1.40*
Instructor encouraged	6.55 ± 1.69	6.12 ± 0.83	5.21 ± 1.44*

## Discussion

In summary, our data demonstrate that physiological intensity, measured with heart rate, and psychological perceptions, assessed with subjective survey responses, were statistically greater for the identical class during the live group condition.

Sustained physical activity patterns reduce the risk of developing noncommunicable diseases and chronic degenerative diseases (12). So, it is also critical to continually gauge what current exercise protocols meet the recommendations with the potential for consistent attendance. Farrance et al. (17) conducted a systematic review of group exercise interventions and concluded that community-based group exercise programs have long-term adherence rates of almost 70%. Kanamori et al. (18) compared the frequency of exercise when individuals exercised alone vs. with others and reported that the frequency of sessions was higher in the group environment. Both the streaming and live formats are group exercise examples that can be completed at home or in an external facility.

Interestingly, live rehabilitation therapy has been practiced for years. Brouwers et al. (19) utilized telerehabilitation, live on demand coaching, as a method to provide improved cardiac therapy in patients with coronary artery disease. They concluded that live internet and video consultations produced sustainable behavioral change which translated into greater cost-effective physical activity habits. Just last year, this strategy was tested by Gagnon et al. (20) in stroke survivors. They reported that the remotely supervised fitness and mobility sessions yielded high levels of retention, improved accessibility, and facilitated engagement due to the structured schedule and social interaction. Similar to our findings, Kenis-Coskun et al. (21) recounted that these live telehealth strategies are superior to non-live video exercise protocols in patients with muscular dystrophy. In short, a live component with an expert is a preferred approach for physical improvement and consistent completion in clinical populations.

Research during the pandemic was valuable to determine the benefits of digital fitness in both streaming as well as on demand formats. Cronshaw (22) piloted a survey study and outlined how physical activity in the home can improve both psychological as well as social well-being through reduced anxiety and enhanced control while feeling connected to an online community. Also through survey data, Liu et al. (23) concluded that digital fitness applications utilizing either live streaming or virtual reality are beneficial tools in promoting and improving health when live scenarios are not feasible. Finally, specific to augmented reality, Ellis et al. (24), reported that these games provide virtual socialization, sustained activity, temporal routine, and mental structure thereby improving both physical and mental health. To sum, digital options are feasible methods to experience the benefits of exercise especially when live options are not available.

Multiple studies focused on understanding individual-level psychological mechanisms such as cognitions, attitudes, and motivations that influence physical activity attendance. However, both investigators and practitioners have struggled to leverage the specific social and environmental variables that promote sustained adherence in physical activity (25). Exercise groups can become the source of affiliation, support, belonging, bonding, and identity that subsequently promote investment (26, 27). Gym and studio settings can feel more like an authentic group when they include: (a) a collective identity, (b) a shared sense of purpose, (c) group structure, and (d) interdependence among members (28). These strong connections with an exercise group predict higher participation in physical activity and exercise-specific satisfaction (25). The benefits are greater in environments that involved strategies to foster member interactions compared to home-based interventions (29). Hence, group exercise scenarios that provide social interaction may have extra physiological, psychological, and adherence rates than exercising alone (30).

Our data also identify a few of the less positive consequences of exercising in a facility outside the home. During the live group classes, participants were more self-conscious and more concerned about their perception. Sabiston and colleagues (31, 32) focused on how self-conscious emotions of shame, guilt, and pride influence physical activity behavior. In short, they concluded that exercise motivation can be negatively impacted by self-conscious feelings. Elliot and Dweck (33) studied perceived competence, an individual's belief that they are capable of being effective at an activity. Although it is not clear how perceived competence influences satisfaction with a class or future adherence, it does facilitate motivation for goal attainment. Together these results illustrate the importance of future studies to create strategies to minimize these negative emotions in a live setting.

With respect to limitations, our sample was fairly homogeneous with respect to race and experience with the experimental class. Consequently, conclusions about the group live class providing an optimal experience can only be generalized to more experienced exercisers. In fact, based on the survey items, less active individuals may find the live streaming class a more ideal combination with the positive group attributes without the negative self-conscious components. Nevertheless, we believe that the findings from this study provide key insights into the differences between formats.

The current study provides evidence that a live group environment is superior to live streaming or on demand fitness formats in terms of high exercise intensity and affirmative subjective feedback. However, in combination with past research, we can conclude that each type has the potential to improve both psychological as well as physiological variables and can therefore be recommended depending on the current needs of the individual. The future phase in this area of research is to assess the adherence component of each format as well as strategies to encourage the quantity and quality of planned exercise.

## Data Availability

The raw data supporting the conclusions of this article will be made available by the authors, without undue reservation.
